# Health Outcomes of Information System Use Lifestyles among Adolescents: Videogame Addiction, Sleep Curtailment and Cardio-Metabolic Deficiencies

**DOI:** 10.1371/journal.pone.0154764

**Published:** 2016-05-05

**Authors:** Ofir Turel, Anna Romashkin, Katherine M. Morrison

**Affiliations:** 1 Department of Information Systems and Decision Sciences, Mihaylo College of Business and Economics, California State University—Fullerton, Fullerton, California, United States of America; 2 Brain and Creativity Institute, Department of Psychology, University of Southern California, Los Angeles, California, United States of America; 3 Department of Pediatrics, McMaster Children Hospital and Faculty of Health Sciences, McMaster University, Hamilton, Ontario, Canada; University of Texas Health Science Center at San Antonio, UNITED STATES

## Abstract

**Background and Objective:**

Obesity is a rising problem among adolescents in modern societies; it results in long-term cardio-metabolic problems. Possible overlooked drivers of obesity and its consequent cardio-metabolic deficits include videogame addiction and the resulting curtailed sleep; both are growing problems among adolescents. The objective of this study is to examine possible associations among these concepts in adolescents, as a means to point to plausible interventions.

**Methods:**

Data were collected from 94 adolescents who play videogames and are enrolled in outpatient clinics, using surveys, wearable sleep monitors (FitBit), physical exams, and blood tests at three points in time. These data were subjected to structural equation modeling (SEM) analyses and bootstrapping-based mediation testing procedures.

**Results:**

Videogame addiction among adolescents was negatively associated with sleep duration (β = -0.24). Sleep duration was negatively associated with obesity (β = -0.30), which in turn was associated with elevated blood pressure (β = 0.26), low high-density lipoprotein cholesterol (β = -0.18), high triglycerides (β = 0.61), and high insulin resistance (β = 0.39). The model explained 36.2% of the variation in sleep duration, 32.7% of the variation in obesity, and between 12.8% and 28.1% of the variation in cardio-metabolic indicators. Post-hoc analyses indicated that curtailed sleep is a possible full mediator of the association between videogame addiction, abdominal obesity and the associated cardio-metabolic deficits.

**Conclusion:**

The findings point to possible information systems use lifestyle-health links, which behooves researchers and practitioners to pay closer attention to possible adverse health outcomes of technology-related addictions. Interventions that target problematic video-gaming and sleep should be devised as a possible means for improving adolescents’ long-term cardio-metabolic health.

## Introduction

This study examines how emerging phenomena in modern societies, such as videogame addiction, curtailed sleep, obesity and cardio-metabolic deficits are related, and specifically how videogame addiction can affect the health of a vulnerable population of adolescents. By doing so it extends and integrates discrete pieces of evidence from prior research into a cohesive nomological network, which explains how curtailed sleep can be a key factor mediating a state related to information system use lifestyle (videogame addiction) and cardio-metabolic health. The objective is to expand the knowledge regarding (1) factors which may lead to long-term cardio-metabolic impairments among adolescents, and (2) the health risks of videogame addiction; this knowledge can lead to increased awareness of such problems as well as to the development of interventions for alleviating these conditions.

Videogame addiction is a state of maladaptive psychological dependency on using videogames, manifested through a pattern of excessive videogame seeking and use behaviors that infringe individuals’ normal functioning and leads to adverse consequences [1]. It is an important phenomenon to understand because: (1) it is fairly prevalent and serious [1, 2]; e.g., it is estimated that between 2% to over 30% of gamers present serious addiction-like symptoms [3], (2) it affects a vulnerable population of adolescents [4, 5], and (3) it can drive major social impairments [4] and health issues [6–13]. Given such issues, it has been recognized as an important topic for further study by professional medical bodies [14] and social science researchers [15].

Obesity, defined as the condition of being over the healthy/recommended weight due to excess fat disposition [16] and consequent poor cardio-metabolic health (i.e., physiological measures associated with heart disease and metabolic disorders such as dysglycemia, hypertension, dyslipidemia and insulin resistance), are growing societal problems. Obesity is common among children and adolescents (individuals ages 2 to 19 years old) [17], and is expected to become even more prevalent in the near future [18]. This is a major concern, as childhood obesity and excess weight increase the risk of hypertension, dyslipidemia [19], and insulin resistance which can lead to Type 2 Diabetes [20]. Moreover, childhood obesity tracks into adulthood [21] and obese children face a greater risk of cardiovascular and coronary diseases as well as Type 2 Diabetes as adults [22, 23]. Therefore, it is urgent to target lifestyle behaviors which are associated with excessive adiposity and its health consequences.

In this study, we propose that videogame addiction is one factor which can be linked to obesity, and that this association is at least partially mediated through sleep curtailment, i.e., reduction in sleep time. Consistent with prior research, we then link obesity to poor cardio-metabolic health indicators. The hypotheses we put forth are based on and integrate several isolated notions and findings, and extend them by shifting focus from screen time effects to addiction effects on sleep (increased screen time is only one possible symptom of addiction out of several which can affect sleep), from the often examined young-adult population to a pediatric population which may be more at risk for engaging in problematic behaviors [24], and from linking addiction and health directly to considering the mediating effects of sleep.

First, we suggest that videogame addiction levels can curtail adolescents' sleep through several processes. Since videogame addiction produces similar symptoms to those observed in the cases of other substance and behavioral addictions [1, 25], it is reasonable to expect that adolescents with high addiction scores will feel strong craving to play videogames, inability to control videogame play time and a constant need to increase this activity; they will also try to avoid unpleasant withdrawal when not playing. This can push adolescents to delay their sleep onset and keep on playing; or merely be preoccupied with playing and find it difficult to fall or stay asleep. In extreme cases, it has been reported that people have died from prolonged videogame playing, delaying sleep onset and avoiding sleep [26]. Another possible reason for the association between addiction and poor sleep may be related to melatonin, which is an important hormone signalling the onset of sleep and need for sleep [11, 12, 27]. Videogame addiction can increase screen time before bed. The light emitted from light-emitting diode (LED) screens emulates day time for the brain, which suppresses the release of melatonin [28–30] and drives reduced sleep [31, 32].

Second, consistent with several meta-analyses [33–35] we propose that curtailed sleep will increase one's levels of obesity. We specifically suggest that short sleep duration might influence abdominal adiposity [36]. Plausible mechanisms explaining this association include increased energy intake (e.g., increased consumption of unhealthy snacks) and reduced energy expenditure (e.g., reduced physical activity) after sleep curtailment [37]. The increase in energy intake can be partially explained by sleep-induced changes in secretion of the appetite hormones leptin and ghrelin. Leptin is a hormone secreted from fat cells that suppresses appetite while ghrelin is predominantly secreted from stomach cells and stimulates appetite. Adult studies report a drop in leptin and a rise in ghrelin levels upon sleep curtailment [38–40] and this dysregulation may cause the increase in hunger and appetite and ultimately weight gain [39, 41–44]. Apart from a physiological change influencing energy intake, sleep curtailment also increases the time available for food consumption [45].

Third, we propose that there may be other effects, beyond the sleep-mediated effect, of videogame addiction on obesity. This can happen through possible increased sedentary time, regardless of sleep [6] and consequent reduced physical activity [46]. Hence, we propose a plausible partially mediated effect of videogame addiction on obesity, through sleep curtailment.

**Fig 1:**
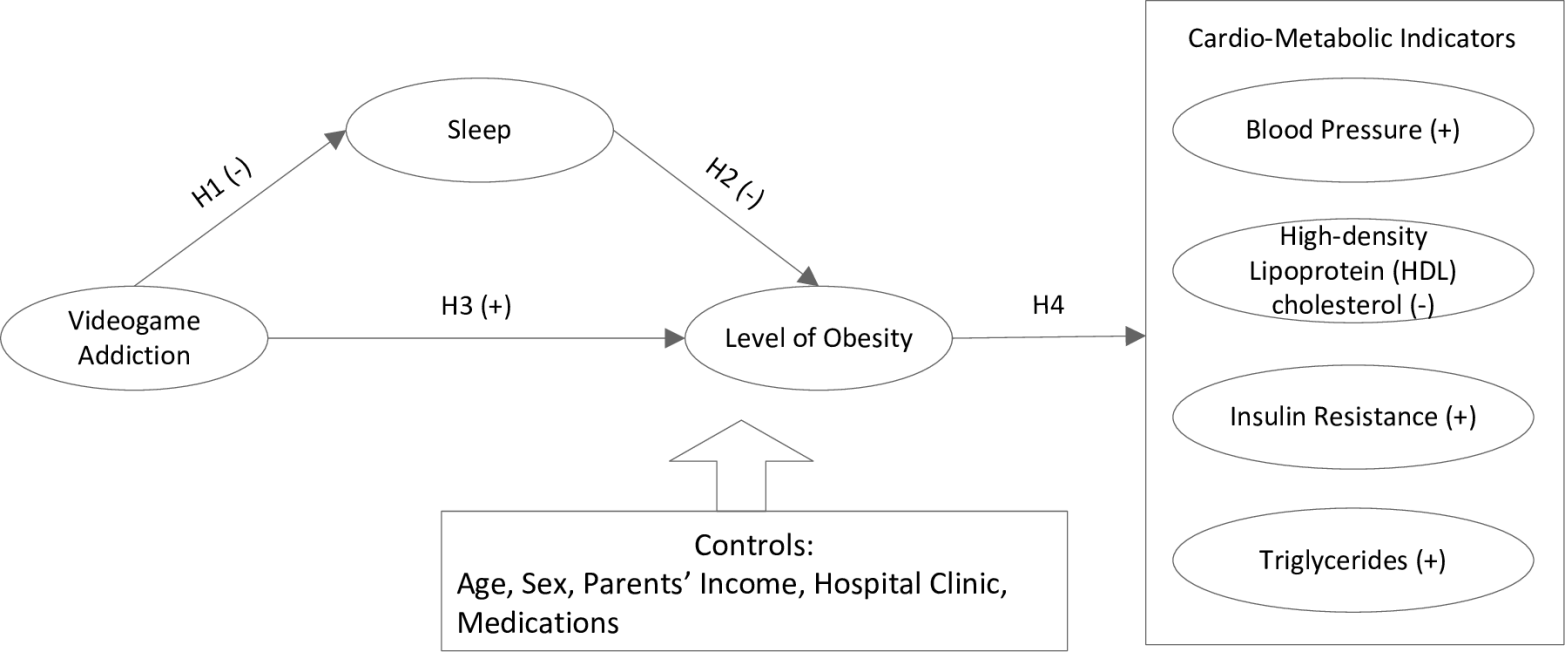
Research Model.

Lastly, obesity can lead to physiological changes in metabolism resulting in dyslipidemia [47], increased blood pressure [48], and insulin resistance. Taken together, our hypotheses and resultant proposed model are portrayed in Fig 1.

## Methods

### Procedure and Sample

The study is a cohort study that included adolescents (10–17 year olds) recruited from two clinics at a large research hospital in North America. The first one was a pediatric lipid clinic which focused primarily on treating children with abnormal lipids, mostly due to genetic reasons. The patients in this clinic were not necessarily obese, but typically had a family history of cholesterol disorders and premature coronary artery disease. The second clinic focused on weight management and typically treated, educated and monitored overweight children, with obesity being the primary referral. The study was approved by McMaster Children's Hospital Research Ethics Board.

Participants were only recruited during the school year in order to reduce the possibility of unusual sleep patterns during breaks. Participants came in with their parents/guardians for a scheduled visit (n = 200), and were introduced to the study by a nurse. Out of these, 157 agreed to participate, but only 125 played videogames (approximately 80% of the consenting sample). Only these individuals were retained in the study. Because the participants were minors, they and their parents/guardians signed assent and consent forms, respectively. After consenting, participants were asked to complete a paper-based survey which captured their levels of videogame addiction, demographic information, and information regarding medication use. Parents were also asked to provide demographic information (annual income) on a separate page and did not intervene in the child’s survey completion.

Next (at the same appointment), participants were given Fitbit devices and were asked to record their sleep for one week, at which point they returned the Fitbit device to the researchers. A demonstration of Fitbit use was performed at this point by the research team. Out of these participants, 94 returned the Fitbit given to them with sleep data recorded (Reasons for not returning sleep duration data included losing the device, damaging the device, forgetting to use the device, and not recording data properly). This corresponds to a 75% response rate from consenting participants, and a total response rate of 47%. Lastly, participants’ blood tests (for measurement of lipids and insulin resistance) and physical measures (waist circumference, height and blood pressure) were taken at a subsequent clinic visit, within one to eight weeks from returning the Fitbits (see depiction of study design in Appendix A in S1 Text).

The final sample included 94 online videogame playing children. Fifty seven (60.6%) came from the weight management clinic, and 37 (39.4%) from the lipid clinic. Sixty three (67%) were males. A few individuals were on medications: five on cholesterol lowering medication (5.3%), four on sleep medications (4.3%), two on blood pressure medications (2.1%), and two on insulin sensitizing medications (2.1%). Possible effects of such factors were controlled for. The samples’ descriptive and anthropometric characteristics are given in Table 1.

**Table 1:** Anthropometric and descriptive characteristics of the subjects.

Characteristics	Range	Average	Standard Deviation
Age (years)	10–17	13.02	2.24
Onset of videogame playing (years of age)	2–14	6.7	2.5
Videogame addiction score	1–4.64	2.29	0.72
Height (cm)	138.3–198	162.0	11.8
Waist circumference (cm)	57–130	89.6	16.9
Waist/Height ratio	0.37–0.74	0.55	0.09
Weight (kg)	36.6–138.3	74.8	23.9
Body fat (%)	3.5–53.3	33.6	12.1
Fasting glucose (mmol/L)	3.4–6.8	4.9	0.45
Fasting insulin (pmol/L)	23–654	125.8	105
Systolic blood pressure (mmHg)	92–146	115.78	10.48
Diastolic blood pressure (mmHg)	53–96	69.59	7.66
Sleep duration (minutes)	329.86–611	484.62	52.21
Triglycerides (mmol/L)	0.39–4.07	1.43	0.80
HDL Cholesterol (mmol/L)	0.31–4.79	1.21	0.55
Insulin resistance (HOMA-IR)	0.66–27.46	4.12	4.06
Annual income of parents (CAD $)	< 49,999 11.7%, 50,000–69,999 22.1%, 70,000–99,999 28.6%, >100,100 37.7%		

### Measures

Two pilot studies were performed in order to validate key measures taken in this study; one for examining the reliability and validity of the addiction scale in adolescent populations, and another for validating the ability of FitBit to accurately measure sleep duration. Both tests supported the viability of the proposed measures (see Appendix B in S1 Text).

#### Videogame addiction

Videogame addiction was captured with the 14 item scale by Van Rooij [25]. This questionnaire captures on a one (Never) to five (Very Often) Likert scale the frequency of addiction symptoms, including salience, withdrawal, conflict, relapse and reinstatement, tolerance and mood modification. The items are included in Appendix C in S1 Text, and were reliable both in the pilot (α = 0.90) and main studies (α = 0.87).

#### Sleep duration

Several options were considered for capturing sleep duration, including parental sleep reporting, polysomnography, and the use of Fitbit. The latter option was chosen for several reasons. First, parental sleep reports are subjective and often suffer from poor parental compliance and inaccurate reporting [49]. Second, while polysomnography is considered the gold standard for objective sleep measurement, it is very expensive, makes it less feasible to measure sleep over multiple days, and it captures sleep in a less natural environment. Considering these disadvantages, the accurate sleep duration measurement of FitBit as demonstrated in the pilot study, and the ability of wearable sleep monitors to accurately and objectively record sleep duration during free style living [50], Fitbit was chosen for this study.

Participants wore the Fitbit for up to one week after the first study visit and were asked to wear it on their non-dominant hand as instructed by Fitbit Inc. Fitbit Ultra was given out to the participants; however with the progression of the study, participants were given the Fitbit One as the Ultra was discontinued. The different Fitbit models were used interchangeably as sleep duration measures from the models were not significantly different (*p* = 0.91). The Fitbit categorized each minute as asleep or awake, which allows the internal calculation of accurate sleep duration, defined as the difference between sleep onset time and wake up time. Participants were instructed to initiate sleep mode by pressing a button on the Fitbit to denote bed time.

#### Physical and cardio-metabolic measures

Participants’ level of central obesity was measured using a ratio of waist circumference to height. This is a common measure of level of obesity, as it captures specifically the presence of the more harmful intra-abdominal fat, i.e., abdominal adiposity [51]. It also accounts for the natural growth in children and for different growth tendencies, by dividing the waist circumference by height, and is fairly stable [52]. Consequently, this measure is advantageous compared to other obesity measures such as Body Mass Index (BMI) and waist circumference. It is better than such measures in predicting and screening for cardio-metabolic risks [53, 54] and in identifying the need for weight management interventions [55] in adults and adolescents [56, 57]. Measures for operationalizing this ratio were taken by a nurse during a clinic visit. Standing height was measured with the subject in bare feet using a Harpenden stadiometer to the nearest 0.5 cm. Waist circumference was measured to the nearest 0.1 cm using a non-stretchable standard tape measure attached to a spring balance. Measurements were done over the unclothed abdomen at the midpoint between the costal margin and the iliac crest.

Blood pressure was captured using an oscillometric device. The systolic and diastolic pressure values were aggregated to a reasonably reliable factor score (α = 0.67) [58, 59].

HDL-C, triglycerides and insulin resistance were recorded from blood drawn in the morning, after a 12 hour fast. HDL-C and triglycerides were measured using an enzymatic colorimetric method on the Roche INTEGRA analyzer [60]. Insulin resistance was measured with the homeostatic model assessment-insulin resistance (HOMA-IR) [61], which is a viable measure since it is highly correlated with the gold standard euglycemic clamp [62]. HOMA-IR was calculated as: [Fasting glucose (mmol/L) x Fasting insulin (μIU/mol) ÷ 22.5]. Fasting glucose was measured with an enzymatic reference method with hexokinase on the Roche INTEGRA analyzer. Fasting insulin was measured using an immunometric Assay on the IMMULITE analyzer [63].

#### Control variables

Several control variables were used. First, given that participants’ physical measures and cardio-metabolic profiles may vary between clinics, clinic type (Weight Management = 0, Lipid = 1) was recorded. Second, demographic information (self-reported by children) such as age and sex can influence some of the variables in the model. For instance, sleep duration often declines with age during adolescence [64], and there can be sex-differences in sleep [65] and obesity [66]. Third, socioeconomic status, operationalized in this study as parents’ income (self-reported by parents on a 1 to 4 Likert scale- from less than $49,000/year to over $100,000/year) can influence obesity levels [67] and cardio-metabolic risks [68]. Lastly, the use of cholesterol lowering, sleep, anti-hypertensive and insulin sensitizing medications (self-reported by children with the help of research staff; Not using = 0, Using = 1) can potentially be associated with this study’s variables [69].

## Results

Correlations among variables and reliability scores for multiple-item constructs are given in Table 2 (Model variables on top and control variables on the bottom).

**Table 2:** Descriptive Statistics, Correlations and Reliability Indices^†^.

	(1)	(2)	(3)	(4)	(5)	(6)	(7)	(8)	(9)	(10)	(11)	(12)	(13)	(14)
(1) Videogame Addiction	.87													
(2) Sleep Duration (minutes)	-.28**	NA												
(3) Abdominal Obesity (no units: cm/cm)	-.01	-.17	NA											
(4) Blood Pressure (mmHg)	-.08	-.16	.27**	.67										
(5) HDL-C (mmol/L)	.16	.15	-.20*	-.17	NA									
(6) Triglycerides (mmol/L)	.10	-.35**	.43**	.20	-.04	NA								
(7) Insulin Resistance (HOMA-IR)	-.01	-.13	.21*	-.04	-.15	.40**	NA							
(8) Age	.13	-.54**	.12	.35**	-.17	.21*	.14	NA						
(9) Sex (Male = 0)	-.29**	.12	.11	-.16	-.05	-.05	.12	-.14	NA					
(10) Parents’ Income	-.09	-.06	.01	-.08	-.17	-.16	.00	-.07	-.03	NA				
(11) Hospital Clinic	.14	-.24*	-.48**	-.05	.06	.08	.18	.25*	-.24*	-.01	NA			
(12) Sleep Medication	.13	.14	.03	.09	-.02	.05	-.02	-.03	-.15	.14	-.06	NA		
(13) Cholesterol Medication	.15	.02	-.11	-.14	.32**	.07	.25*	-.00	-.17	-.02	.29**	-.05	NA	
(14) Blood Pressure Medication	-.05	.02	.01	.41**	-.04	-.05	-.01	-.00	-.10	-.16	-.12	-.03	-.04	NA
(15) Insulin Sensitization Medication	.07	.05	.01	.02	-.00	.00	.01	-.10	.05	.02	-.12	-.03	-.03	-.02

† On the diagonal: Cronbach’s Alpha for multiple-item constructs

* *p* < 0.05

** *p* < 0.01

Next, we wanted to ensure that the sample is viable for testing the proposed model with structural equation modeling techniques. Even though such models with sample sizes over 50 tend to generate correct solutions with acceptably low non-convergence rates [70], and key fit indices are not that sensitive to sample size, e.g., sample size explains <1% of the variation in the Comparative Fit Index (CFI) and the Root Mean Square Error of Approximation (RMSEA) [70], there is still the risk that our model is not sufficiently powered to reject null hypotheses. Hence, the minimum sample size required for obtaining a statistical power of 0.90 (with ε = 0.10 and for α = 0.05) for RMSEA for a model with 38 degrees of freedom was calculated using critical non-centrality parameters [71]. The minimum sample was 85, and the sample used in this study was larger. It was therefore concluded that it was adequate for SEM path analyses.

**Fig 2:**
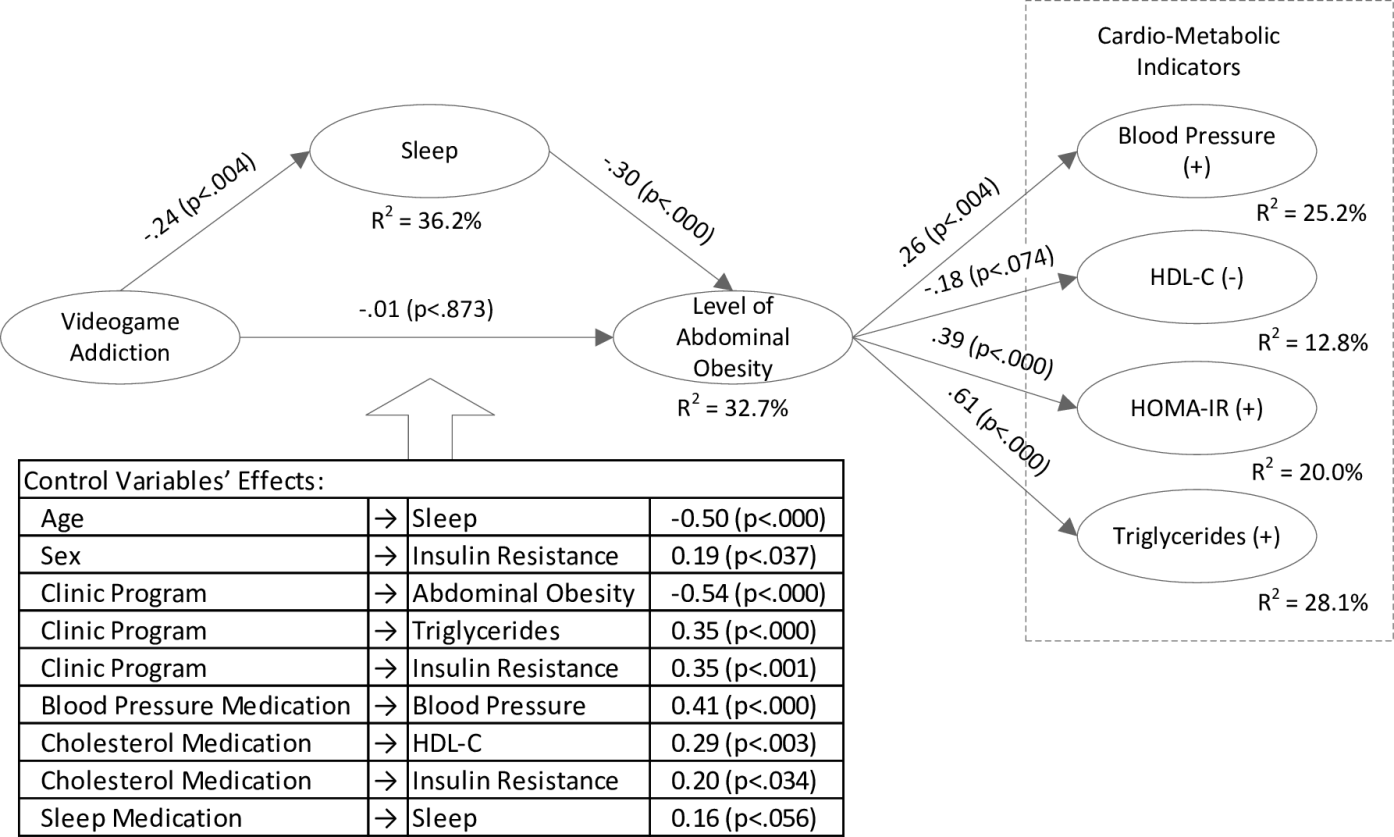
Structural Model.

Lastly, the hypothesized model was estimated with the SEM facilities of AMOS 23. Initially, we included all the control variables in the model. All hypothesized effects were significant (at least p<0.10) except for the direct path from videogame addiction to obesity, and the model had good fit [χ^2^(9) = 12.9, p<0.17; CFI = 0.98; IFI = 0.99; GFI = 0.98; SRMR = 0.023; RMSEA = 0.068 (p-close = 0.31)]. However, several control variables did not significantly influence at least several of the model’s variables. For parsimony reasons these paths were removed and the model was re-estimated. It had good fit: χ^2^(36) = 39.00, p<0.31; CFI = 0.98; IFI = 0.99; GFI = 0.94; SRMR = 0.062; RMSEA = 0.030 (p-close = 0.68). The standardized path coefficients, their levels of significance, and the variance explained in the endogenous variables are given in Fig 2. The model explains 36% of the variation in sleep, almost one-third of the variation in abdominal obesity, and between 13.1% and 28.1% of the variance in users’ cardio-metabolic health indicators.

### Post-hoc Analyses

First, the findings imply that adolescents’ sleep fully mediates the effect of videogame addiction on obesity. This indirect effect was tested with the bias-corrected bootstrapping procedure outlined by Cheung and Lau [72] using Amos 23. The bootstrapping procedure is advantageous to the Sobel test and alike, since the product of two parameters is not normally distributed [72]. The results of this procedure show that the indirect effect was significant: lower bound = .02, upper bound = .16, p < .014. At the same time, the direct effect of videogame addiction on obesity was not significant: lower bound = -0.18, upper bound = 0.17, p<0.96. Hence, full mediation was demonstrated and it appears that sleep curtailment is a key mechanism which may translate videogame addiction into obesity.

Second, our sample included very few patients who were receiving medications. While we controlled and accounted for the possible effects of medications, we also tested if the results still hold after removing the medicated patients from the sample (and removing corresponding controls from the model). The model presented good fit: χ^2^(21) = 26.19, p<0.19; CFI = 0.97; IFI = 0.97; GFI = 0.94; SRMR = 0.077; RMSEA = 0.055 (p-close = 0.41). All hypothesized paths, except for the one from addiction to obesity, were significant: Standardized β_Addiction→Sleep_ = -.25, p < .006; β_Sleep→obesity_ = -.32, p < .000; β_Addiction→obesity_ = -.02, p < .848; β_Obesity→HDL_ = -.32, p < .003; β_Obesity→Triglycerides_ = .55, p < .000; β_Obesity→Insulin Resistance_ = .31, p < .009; β_Obesity→Blood Pressure_ = .29, p < .002. This model explained 35% of the variance in sleep duration and obesity, and 10% to 25% in the variance in cardio-metabolic indicators.

Third, in order to further lend support to the proposed association, the data were stratified into two groups of the bottom 45 (0.371 to 0.557) and highest 45 (0.561 to 0.742) abdominal adiposity scores. Mean videogame addiction and sleep duration were calculated in each stratum (See Table 3). As can be seen, individuals in the low obesity stratum presented lower videogame addiction and higher sleep duration than did individuals in the high obesity stratum.

**Table 3:** Means based on Stratified Abdominal Adiposity.

	Bottom 45	Top 45
Videogame Addiction	2.28 (SD = 0.73)	2.35 (SD = 0.71)
Sleep Duration	491.1 (SD = 48.5)	476.6 (SD = 57.1)

## Discussion

This study sought to examine if and how videogame addiction can be negatively associated with adolescents’ health, as captured by their levels of abdominal obesity and a range of consequent cardio-metabolic impairments. The findings based on data from a sample of 94 adolescents (M_Weight_ = 74.8kg, M _Waist circumference_ = 89.6cm, M_Body fat_ = 33.6%, M_HOMA-IR_ = 4.12) show that sleep curtailment is a possible mechanism that mediates the association between videogame addiction and abdominal obesity; and confirms the relationship between abdominal obesity and low HDL-C levels and high levels of triglycerides, blood pressure and insulin resistance. By doing so, this study demonstrates and explains technology addiction-related health risks among adolescents and the plausible role of sleep curtailment in this process; it integrates addiction, sleep and obesity research into a single nomological network and extends knowledge from the young-adult and adult domains to the domain of adolescents.

Specifically, this study introduces curtailed sleep as an important possible outcome of technology addiction and perhaps other IS use phenomena. Sleep is an important health factor [36], which has the potential to be influenced, as demonstrated in this study, by technology-related addictions. Sleep curtailment affects people’s health and functioning and influences adolescent obesity [34]. This study brings this important, yet relatively unexplored, issue to the limelight. Given the adverse consequences of curtailed sleep demonstrated in this study, we call for further integration of sleep or sleep curtailment into models focusing on adolescent lifestyle, and specifically adolescent IS use patterns and technology-related addictions.

Furthermore, this study informs the literature on technology-related addictions in at least two ways. First, the existing body of work has focused on a range of negative outcomes, including psychological wellbeing of users, school or work performance, and conflicts with family and friends [73]. It has conceptually pointed to potential health implications of such addictions [74], but has largely provided little theoretical development regarding such associations and proof regarding their existence. This study provides such theory development and evidence, and paves the way for further studying sleep and cardio-metabolic health issues indirectly associated with various aspects of problematic IS use. Second, much of the work on technology addictions was done with samples from young-adult populations [73]. This study adds to the limited set of studies focusing on adolescent populations (e.g., [1]). These populations are more at risk than others for engaging in risky behaviors, developing addictions and long term damage [75]. Hence we call for further studies of this vulnerable population and the long term effects of early onset of IS use on it.

Moreover, this study further extends the arsenal of tools researchers can employ, by introducing an efficient and accurate way to measure sleep, i.e., with inexpensive wearable sleep monitors such as Fitbit. Sleep measurement has been a cumbersome and challenging task, and with the use of tools such as FitBit, it can become more manageable.

Given the important relationships between abdominal obesity and adverse cardio-metabolic health consequences, it is important for medical researchers to identify modifiable behaviours and psychological states that may influence abdominal obesity. This study suggests that it may be possible to improve cardio-metabolic health among adolescents through prevention or reduction of videogame addiction and through normalization of adolescents’ sleep time; and these factors seem to be associated. Interventions for controlling videogame play time may include increased awareness among children, parents, physicians and educators, and possibly the use of educational videos about the risks of videogame addiction [76]. This may help not only in prevention, but also in early detection of videogame addiction problems. Similarly, increased awareness of parents, physicians and children regarding sleep curtailment effects may help children better regulate their sleep, though this requires further research.

Lastly, it is interesting to consider whether and how the IS developers and researchers community can improve adherence to common codes of conducts [77, 78], which often call for IS professionals to try to minimize the threats of the applications they develop to the health of users. This is not to say that IS professionals are to be blamed for addiction and obesity issues in society. Rather, it is hoped that this study serves as a reminder regarding potential health harms of technology addictions, and a call to be more mindful regarding potential health risks of certain applications.

Several limitations of this study should be taken into account. First, this study was conducted with adolescents who were enrolled in specific clinics. As such, our findings may not be applicable to the broader adolescent population, and we call for replication studies with different populations. Second, this study focused on one key relatively stable predictor of curtailed sleep, video game addiction. There are many other possible stable (e.g., parental oversight, chronic stress) and situational (e.g., flu, situational stress) predictors of sleep. Hence, future research can incorporate more predictors of sleep. Similarly, while we focused on sleep and several common control variables as predictors of obesity and cardio-metabolic health, there may be many other factors, e.g., genetics and environmental factors, affecting them. Future research can therefore extend our model and include such factors. Third, we captured each variable at one point in time. While medical findings and theory support the proposed association between the time-lagged data we obtained, future studies employing longitudinal or experimental designs can further support the proposed model and especially the causality arguments which cannot be fully supported in cross-sectional designs. Fourth, we assumed direct effects between our variables, even though there may be many nuanced mechanisms, including hormonal processes pertaining to cortisol, melatonin and leptin, which mediate these effects. For example, light exposure may stimulate the pineal gland and through this mechanism, among many others, videogame addiction can influence sleep. In addition, sedentary time, physical activity habits and state of training (e.g., measured with cardiopulmonary exercise test) may possibly mediate associations between videogame addiction and obesity. We call for future research to examine such nuanced processes, account for the underlying hormonal mechanisms which we allude to in this study, yet do not measure, and possibly compare people with low and high addiction scores in terms of such mediating variables.

## Conclusion

Childhood obesity and cardio-metabolic disturbances are of increasing concern. The possible roles of videogame addiction and curtailed sleep in shaping these problems have thus far been largely overlooked. The findings lend support to the idea that videogame addiction is one possible indirect driver of poor cardio-metabolic health. Specifically, videogame addiction can be associated with increased sleep curtailment which in turn can be associated with elevated abdominal adiposity and its resultant cardio-metabolic impairments. It is hoped that our findings pave the way for further research on the health outcomes of technology addictions and possibly other IS use lifestyle variables and on possible interventions that can help protecting vulnerable populations such as children from harms brought by problematic use of technologies.

## Supporting Information

S1 Text Study Design **(Appendix A).** Pilot Tests **(Appendix B).** Videogame Addiction Scale **(Appendix C).**
(DOCX)

## References

[1] Xu ZC, Turel O, Yuan YF. Online game addiction among adolescents: motivation and prevention factors. European Journal of Information Systems. 2012;21(3):321–40. 10.1057/ejis.2011.56 .

[2] Weinstein A. Internet and videogame addiction and the neurobiological basis of behavioral addictions. Journal of Behavioral Addictions. 2013;2:5–6. .

[3] Sepehr S, Head M, editors. Online Video Game Addiction: A Review and an Information Systems Research Agenda. Proceedings of the Nineteenth Americas Conference on Information Systems; 2013; Chicago, Illinois: AIS.

[4] Gentile D. Pathological Video-Game Use Among Youth Ages 8 to 18: A National Study. Psychological Science. 2009;20(5):594–602. 10.1111/j.1467-9280.2009.02340.x 19476590

[5] Le Heuzey MF, Mouren MC. Videogame addiction: a danger for only at-risk children or for all children. Bulletin De L Academie Nationale De Medecine. 2012;196(1):15–23. . 23259329

[6] Fullerton S, Taylor AW, Dal Grande E, Berry N. Measuring physical inactivity: do current measures provide an accurate view of "sedentary" video game time? Journal of obesity. 2014;2014:287013–. 10.1155/2014/287013 . 25002974 PMC4066947

[7] Ivarsson M, Anderson M, Akerstedt T, Lindblad F. The Effect of Violent and Nonviolent Video Games on Heart Rate Variability, Sleep, and Emotions in Adolescents With Different Violent Gaming Habits. Psychosomatic Medicine. 2013;75(4):390–6. 10.1097/PSY.0b013e3182906a4c . 23645706

[8] Lyons EJ, Tate DF, Ward DS, Wang X. Energy intake and expenditure during sedentary screen time and motion-controlled video gaming. American Journal of Clinical Nutrition. 2012;96(2):234–9. 10.3945/ajcn.111.028423 . 22760571 PMC3396440

[9] Scharrer E, Zeller A. Active and Sedentary Video Game Time Testing Associations With Adolescents' BMI. Journal of Media Psychology-Theories Methods and Applications. 2014;26(1):39–49. 10.1027/1864-1105/a000109 .

[10] Vandewater EA, Shim MS, Caplovitz AG. Linking obesity and activity level with children's television and video game use. Journal of Adolescence. 2004;27(1):71–85. 10.1016/j.adolescence.2003.10.003 . 15013261

[11] Weaver E, Gradisar M, Dohnt H, Lovato N, Douglas P. The Effect of Presleep Video-Game Playing on Adolescent Sleep. Journal of Clinical Sleep Medicine. 2010;6(2):184–9. . 20411697 PMC2854707

[12] Wolfe J, Kar K, Perry A, Reynolds C, Gradisar M, Short MA. Single night video-game use leads to sleep loss and attention deficits in older adolescents. Journal of Adolescence. 2014;37(7):1003–9. 10.1016/j.adolescence.2014.07.013 . 25118041

[13] Yamamoto S. Adverse effects of video display terminals on health. Asian Medical Journal. 1999;42(6):245–52.

[14] American Psychiatric Association. Internet Gaming Disorder Diagnostic and statistical manual of mental disorders (5th ed). 5th ed. Arlington, VA: American Psychiatric Publishing; 2013 p. 795–8.

[15] Tarafdar M, D'Arcy J, Turel O, Gupta A. The dark side of information technology. MIT Sloan Management Review. 2015;56(2 (Winter)):600–23.

[16] Must A, Strauss RS. Risks and consequences of childhood and adolescent obesity. International Journal of Obesity. 1999;23:S2–S11. 10.1038/sj/ijo/0800852 . 10340798

[17] Monteiro POA, Victora CG. Rapid growth in infancy and childhood and obesity in later life—a systematic review. Obesity Reviews. 2005;6(2):143–54. 10.1111/j.1467-789X.2005.00183.x . 15836465

[18] Marques A, De Matos MG. Trends and Correlates of Overweight and Obesity Among Adolescents from 2002 to 2010: A Three-Cohort Study Based on a Representative Sample of Portuguese Adolescents. American Journal of Human Biology. 2014;26(6):844–9. 10.1002/ajhb.22613 . 25176416

[19] Weiss R, Dziura J, Burgert TS, Tamborlane WV, Taksali SE, Yeckel CW, et al Obesity and the metabolic syndrome in children and adolescents. New England Journal of Medicine. 2004;350(23):2362–74. 10.1056/NEJMoa031049 . 15175438

[20] Steinberger J, Daniels SR. Obesity, Insulin Resistance, Diabetes, and Cardiovascular Risk in Children. Circulation. 2003:1448–53. 12642369 10.1161/01.cir.0000060923.07573.f2

[21] Whitaker RC, Wright JA, Sepe MS, Seidel KD, Dietz WH. Predicting Obesity in Young Adulthood From Childhood and Parental Obesity. The New England Journal of Medicine. 1997:869–73. 10.1056/NEJM199709253371301 9302300

[22] Reilly J, Kelly J. Long-term impact of overweight and obesity in childhood and adolescence on morbidity and premature mortality in adulthood: systmatic review. International Journal of Obesity. 2011:891–8. 10.1038/ijo.2010.222 20975725

[23] Tirosh A, Shai I, Afek A, Dubnov-Raz G, Ayalon N, Gordon B, et al Adolescent BMI Trajectory and Risk of Diabetes versus Coronoary Disease. The New England Journal of Medicine. 2011:1315–25. 10.1056/NEJMoa1006992 PMC4939259 21470009

[24] Casey BJ, Giedd JN, Thomas KM. Structural and functional brain development and its relation to cognitive development. Biological Psychology. 2000;54(1–3):241–57. 10.1016/s0301-0511(00)00058-2 . 11035225

[25] van Rooij AJ, Schoenmakers TM, Vermulst AA, van den Eijnden R, van de Mheen D. Online video game addiction: identification of addicted adolescent gamers. Addiction. 2011;106(1):205–12. 10.1111/j.1360-0443.2010.03104.x . 20840209

[26] Hunt K, Ng N. Man dies in Taiwan after 3-day online gaming binge. CNN [Internet]. 2015 Jan. 21, 2015. Available: http://edition.cnn.com/2015/01/19/world/taiwan-gamer-death/.

[27] King DL, Gradisar M, Drummond A, Lovato N, Wessel J, Micic G, et al The impact of prolonged violent video-gaming on adolescent sleep: an experimental study. Journal of Sleep Research. 2013;22(2):137–43. 10.1111/j.1365-2869.2012.01060.x . 23137332

[28] Cajochen C, Frey S, Anders D, Spati J, Bues M, Pross A, et al Evening exposure to a light-emitting diodes (LED)-backlit computer screen affects circadian physiology and cognitive performance. Journal of Applied Physiology. 2011;110(5):1432–8. 10.1152/japplphysiol.00165.2011 . 21415172

[29] West KE, Jablonski MR, Warfield B, Cecil KS, James M, Ayers MA, et al Blue light from light-emitting diodes elicits a dose-dependent suppression of melatonin in humans. Journal of Applied Physiology. 2011;110(3):619–26. 10.1152/japplphysiol.01413.2009 . 21164152

[30] Wood B, Rea MS, Plitnick B, Figueiro MG. Light level and duration of exposure determine the impact of self-luminous tablets on melatonin suppression. Applied Ergonomics. 2013;44(2):237–40. 10.1016/j.apergo.2012.07.008 . 22850476

[31] Drescher AA, Goodwin JL, Silva GE, Quan SF. Caffeine and Screen Time in Adolescence: Associations with Short Sleep and Obesity. Journal of Clinical Sleep Medicine. 2011;7(4):337–42. 10.5664/jcsm.1182 . 21897768 PMC3161764

[32] Magee CA, Lee JK, Vella SA. Bidirectional Relationships Between Sleep Duration and Screen Time in Early Childhood. Jama Pediatrics. 2014;168(5):465–70. 10.1001/jamapediatrics.2013.4183 . 24589672

[33] Cappuccio FP, Taggart FM, Kandala N-B, Currie A, Peile E, Stranges S, et al Meta-analysis of Short Sleep Duration and Obesity in Children and Adults. Sleep. 2008:619–26. 18517032 10.1093/sleep/31.5.619 PMC2398753

[34] Marshall NS, Glozier N, Grunstein RR. Is sleep duration related to obesity? A critical review of the epidemiological evidence. Sleep Medicine Reviews. 2008;12(4):289–98. 10.1016/j.smrv.2008.03.001 . 18485764

[35] Xiaoli C, Beydoun MA, Wang Y. Is Sleep Duration Association With Childhood Obesity? A Systematic Review and Meta-analysis. Obesity. 2008:265–74. 10.1038/oby.2007.63 18239632

[36] Chaput J-P, Tremblay A. Does short sleep duration favor abdominal adiposity in children? International Journal of Pediatric Obesity. 2007:188–91. 17999284 10.1080/17477160701306144

[37] Taheri S. The link between short sleep duration and obesity: we should recommend more sleep to prevent obesity. Arch Dis Child. 2006:881–4. 17056861 10.1136/adc.2005.093013 PMC2082964

[38] Mullington J, Chan J, Van Dongen H, Szuba M, Samaras J, Price N, et al Sleep Loss Reduces Diurnal Rhythm Amplitude of Leptin in Healthy Men. Journal of Neuroendocrinology. 2003:851–4. 12899679 10.1046/j.1365-2826.2003.01069.x

[39] Spiegel K, Tasali E, Penev P, Van Cauter E. Brief communication: Sleep curtailment in healthy young men is associated with decreased leptin levels, elevated ghrelin levels, and increased hunger and appetite. Annals of Internal Medicine. 2004;141(11):846–50. . 15583226 10.7326/0003-4819-141-11-200412070-00008

[40] Taheri S, Lin L, Austin D, Young T, Mignot E. Short Sleep Duration is Associated with Reduced Leptin, Elevated Ghrelin, and Increased Body Mass Index. PLoS Medicine. 2004:210–7. 10.1371/journal.pmed.0010062 PMC535701 15602591

[41] Spiegel K, Knutson K, Leproult R, Tasali E, Van Cauter E. Sleep loss: a novel risk factor for insulin resistance and Type 2 diabetes. Journal of Applied Physiology. 2005;99(5):2008–19. 10.1152/japplphysiol.00660.2005 . 16227462

[42] Spiegel K, Leproult R, L'Hermite-Baleriaux M, Copinschi G, Penev PD, Van Cauter E. Leptin levels are dependent on sleep duration: Relationships with sympathovagal balance, carbohydrate regulation, cortisol, and thyrotropin. Journal of Clinical Endocrinology & Metabolism. 2004;89(11):5762–71. 10.1210/jc.2004-1003 . 15531540

[43] Spiegel K, Leproult R, Van Cauter E. Impact of sleep debt on metabolic and endocrine function. Lancet. 1999;354(9188):1435–9. 10.1016/s0140-6736(99)01376-8 . 10543671

[44] Spiegel K, Tasali E, Leproult R, Van Cauter E. Effects of poor and short sleep on glucose metabolism and obesity risk. Nature Reviews Endocrinology. 2009;5(5):253–61. 10.1038/nrendo.2009.23 . PMC4457292 19444258

[45] Chaput J-P, Klingenberg L, Sjodin A. Do all sedentary activities lead to weight gain: sleep does not. Curr Opin Clin Nutr Metab Care. 2010:601–7. 10.1097/MCO.0b013e32833ef30e 20823775

[46] Matheson DM, Killen JD, Wang Y, Varady A, Robinson TN. Children's food consumption during television viewing. American Journal of Clinical Nutrition. 2004;79(6):1088–94. . 15159240 10.1093/ajcn/79.6.1088

[47] Dattilo AM, Krisetherton PM. Effects of weight-reduction on blood-lipids and lipoproteins: A metaanalysis. American Journal of Clinical Nutrition. 1992;56(2):320–8. . 1386186 10.1093/ajcn/56.2.320

[48] Chiba Y, Saitoh S, Takagi S, Ohnishi H, Katoh N, Ohata J, et al Relationship between visceral fat and cardiovascular disease risk factors: The Tanno and Sobetsu study. Hypertension Research. 2007;30(3):229–36. 10.1291/hypres.30.229 . 17510504

[49] Sadeh A. Commentary: Comparing Actigraphy and Parental Report as Measures of Children's Sleep. Journal of Pediatric Psychology. 2008:406–7. 10.1093/jpepsy/jsn018 18310663

[50] Ancoli-Israel S, Cole R, Alessi C, Chambers M, Moorcrof W, Pollak CP. The Role of Actigraphy in the Study of Sleep and Circadian Rhythms. Sleep. 2003:342–92. 12749557 10.1093/sleep/26.3.342

[51] Ashwell M, Cole TJ, Dixon AK. Ratio of waist circumference to height is strong predictor of intra-abdominal fat. British Medical Journal. 1996;313(7056):559–60. . 10.1136/bmj.313.7056.559d PMC2351911 8790002

[52] Sung RYT, So HK, Choi KC, Nelson EAS, Li AM, Yin JAT, et al Waist circumference and waist-to-height ratio of Hong Kong Chinese children. Bmc Public Health. 2008;8 10.1186/1471-2458-8-324 . PMC2563004 18808684

[53] Ashwell M, Gunn P, Gibson S. Waist-to-height ratio is a better screening tool than waist circumference and BMI for adult cardiometabolic risk factors: systematic review and meta-analysis. Obesity Reviews. 2012;13(3):275–86. 10.1111/j.1467-789X.2011.00952.x . 22106927

[54] Savva SC, Tornaritis M, Savva ME, Kourides Y, Panagi A, Silikiotou N, et al Waist circumference and waist-to-height ratio are better predictors of cardiovascular disease risk factors in children than body mass index. International Journal of Obesity. 2000;24(11):1453–8. 10.1038/sj.ijo.0801401 . 11126342

[55] Ashwell M, Lejeune S, McPherson K. Ratio of waist circumference to height may be better indicator of need for weight management. British Medical Journal. 1996;312(7027):377–. . 10.1136/bmj.312.7027.377 PMC2350287 8611847

[56] Khoury M, Manlhiot C, McCrindle BW. Role of the Waist/Height Ratio in the Cardiometabolic Risk Assessment of Children Classified by Body Mass Index. Journal of the American College of Cardiology. 2013;62(8):742–51. 10.1016/j.jacc.2013.01.026. 10.1016/j.jacc.2013.01.026 23500256

[57] Taylor RW, Jones IE, Williams SA, Goulding A. Evaluation of waist circumference, waist-to-hip ratio, and the conicity index as screening tools for hight trunk fat mass, as measured by dual-energy X-ray absorptiometry, in children aged 3–19 y. Am J Clin Nutr. 2007:490–5. 10.1093/ajcn/72.2.490 10919946

[58] Boutouyrie P, Bussy C, Lacolley P, Girerd X, Laloux B, Laurent S. Association between local pulse pressure, mean blood pressure, and large-artery remodeling. Circulation. 1999;100(13):1387–93. . 10500038 10.1161/01.cir.100.13.1387

[59] Ravid M, Brosh D, Ravid-Safran S, Levy Z, Rachmani R. Main risk factors for nephropathy in type 2 diabetes mellitus are plasma cholesterol levels, mean blood pressure, and hyperglycemia. Archives of Internal Medicine. 1998;158(9):998–1004. 10.1001/archinte.158.9.998 . 9588433

[60] Michigan Diabetes Research and Training Center. MDRTC Lipid Measurement Fact Sheet Ann Harbor, MI: University of Michigan Health System; 2014 [cited 2014 Nov. 12]. Available: http://www.med.umich.edu/borc/cores/ChemCore/lipids.htm.

[61] Ferreira AP, Oliveira CER, Franca NM. Metabolic syndrome and risk factors for cardiovascular disease in obese children: the relationship with insulin resistance (HOMA-IR). Jornal De Pediatria. 2007;83(1):21–6. 10.2223/jped.1562 . 17183416

[62] Singh B, Saxena A. Surrogate markers of insulin resistance: A review. World Journal of Diabetes. 2010:36–47. 10.4239/wjd.v1.i2.36 21537426 PMC3083884

[63] Lee JM, Okumura MJ, Davis MM, Herman WH, Gurney JG. Prevalence and Determinants of Insulin Resistance Among U.S. Adolescents. Diabetes Care. 2006:2427–32. 17065679 10.2337/dc06-0709

[64] Van Cauter E, Leproult R, Plat L. Age-related changes in slow wave sleep and REM sleep and relationship with growth hormone and cortisol levels in healthy men. Jama-Journal of the American Medical Association. 2000;284(7):861–8. 10.1001/jama.284.7.861 . 10938176

[65] Dijk DJ, Beersma DGM, Bloem GM. Sex-differences in sleep EEG of young adults: Visual scoring and spectral-analysis. Sleep. 1989;12(6):500–7. . 2595173 10.1093/sleep/12.6.500

[66] Lovejoy JC, Sainsbury A, Stock Conf Working G. Sex differences in obesity and the regulation of energy homeostasis. Obesity Reviews. 2009;10(2):154–67. 10.1111/j.1467-789X.2008.00529.x . 19021872

[67] Sobal J, Stunkard AJ. Socioeconomic status and obesity: A review of the literature. Psychological Bulletin. 1989;105(2):260–75. 10.1037/0033-2909.105.2.260 . 2648443

[68] Staiano AE, Harrington DM, Barreira TV, Katzmarzyk PT. Sitting time and cardiometabolic risk in US adults: associations by sex, race, socioeconomic status and activity level. British Journal of Sports Medicine. 2014;48(3):213–9. 10.1136/bjsports-2012-091896 . 23981954 PMC4019392

[69] Rappley M, Luo Z, Brady J, Gardiner J. Variation in the use of sleep medication for children. Journal of Developmental and Behavioral Pediatrics. 2003;24(5):394–. .

[70] Fan X, Thompson B, Wang. L. Effects of sample size, estimation methods, and model specification on structural equation modeling fit indexes. Structural Equation Modeling: a Multidisciplinary Journal. 1999;6(1):56–83.

[71] Kim KH. The relation among fit indexes, power, and sample size in structural equation modeling. Structural Equation Modeling-a Multidisciplinary Journal. 2005;12(3):368–90. .

[72] Cheung GW, Lau RS. Testing mediation and suppression effects of latent variables—Bootstrapping with structural equation models. Organizational Research Methods. 2008;11(2):296–325. 10.1177/104428107300343 .

[73] Byun S, Ruffini C, Mills JE, Douglas AC, Niang M, Stepchenkova S, et al Internet addiction: Metasynthesis of 1996–2006 quantitative research. Cyberpsychology & Behavior. 2009;12(2):203–7. 10.1089/cpb.2008.0102 . 19072075

[74] Turel O, Serenko A, Giles P. Integrating technology addiction and use: An empirical investigation of online auction sites. MIS Quarterly. 2011;35(4):1043–61. .

[75] Kuss DJ, Griffiths MD, Binder JF. Internet addiction in students: Prevalence and risk factors. Computers in Human Behavior. 2013;29(3):959–66. 10.1016/j.chb.2012.12.024.

[76] Turel O, Mouttapa M, Donato E. Preventing problematic Internet use through video-based interventions: a theoretical model and empirical test. Behaviour & Information Technology. 2015;34(4):349–62. 10.1080/0144929X.2014.936041

[77] Anderson RE, Johnson DG, Gotterbarn D, Perrolle J. Using the new ACM code of ethics in decision making. Communications of the ACM. 1993;36(2):98–107. 10.1145/151220.151231 .

[78] Australian Computer Society. ACS Code of Professional Conduct, V.2.1. Sydney, Australia: Professional Standards Board, 2014.

