# Supplementary material for: Health Outcomes of Information System Use Lifestyles among Adolescents: Videogame Addiction, Sleep Curtailment and Cardio-Metabolic Deficiencies
Source: PLoS One. 2016 May 5;11(5):e0154764. doi: 10.1371/journal.pone.0154764 (PMC4858285; doi:10.1371/journal.pone.0154764)
Supplement: S1 Text — Study Design (Appendix A). Pilot Tests (Appendix B). Videogame Addiction Scale (Appendix C). (DOCX) [file pone.0154764.s001.docx]

# **S1 Text. Appendices- Health Outcomes of Information System Use Lifestyles among Adolescents: Videogame Addiction, Sleep Curtailment and Cardio-metabolic Deficiencies**

# **S1 Text. Appendix A - Study Design**

# **S1 Text. Appendix B - Pilot Tests**

Two pilot tests were carried out in order to validate key measures taken in this study. First, the survey instrument’s reliability and validity in adolescent populations as well as some of our assumptions regarding potential mechanisms linking addiction to sleep curtailment were examined. Second, the ability of FitBit to accurately measure sleep duration was tested by comparing its measures with those produced by a well-established sleep measurement device (ActiSleep monitor).

*Pilot testing the Survey*

The survey instrument (including several validity check items) was given to 32 adolescents (9-17 years old, average age=13) who play videogames for assessing their ability to comprehend it and to check several assumptions regarding videogame addiction. Based on participants’ feedback and interactions with the supervising nurse, it appeared that regardless of age, participants could comprehend the survey. It further demonstrated that the addiction measure taps into relevant symptoms which participants have occasionally experienced (average addiction score=2.38 on a 1-5 scale). In addition, the addiction scale was reliable and valid both for the whole sample as well as when the sample is split into younger (age<=12) and older (age>=13) adolescents (Cronbach alpha = 0.90 with significant high loadings [λ>0.59, p<0.001] - both for the full and sub-samples). The split by age group also indicated that there were no significant differences in the addiction scores by age group (F_1,30_=0.47, p<0.50), and no differences in number of missing values (none in both groups). Evan at a symptom-by-symptom level, there was no omnibus difference between the age groups (Pillai’s Trace =0.496, p<0.36). Hence, it was concluded that younger respondents did not differ in their average levels of addiction and symptoms, as well as in the reliability and validity of responses from older ones. This reaffirms the ability of this survey instrument to be used with adolescents, as demonstrated in past research [[1](#_ENREF_1)].

Lastly, to check whether our assumptions regarding the possible mechanisms linking addiction to sleep curtailment hold, the pilot study captured (1) two variables indicating whether it is common for participants to play videogames right before bedtime until they fall asleep, and whether they typically play videogames between 4pm to 8pm, and (2) three variables capturing videogame use (days per week and hours per day they play videogames as well as typical videogame session duration). Addiction scores were positively correlated with videogame play before bed time (r=0.37, p<0.05) and in the 4pm to 8pm timeslot (r=0.35, p<0.05), which is close to many adolescents’ bed time. Addiction scores also positively correlated with video-gaming frequency (r=0.51, p<0.01), daily hours of videogames (r=0.38, p<0.05), and the duration of typical video-gaming sessions (r=0.32, p<0.05). Furthermore, a logistic regression model indicated that videogame addiction is a significant predictor of videogame play right before falling asleep (β=1.96, p<0.05; pseudo Nagelkerle r-square of 30%). In other words, an increase of one point in one’s addiction score translates into a 7.1% increase in chances for them to play videogames right before bed. These findings support the notions we propose in the text, that videogame addiction can be associated with curtailed sleep through various mechanisms, including increased videogame play, lengthy play sessions, and increased playtime before going to bed. The 30% explained variance implies that there are other possible factors (e.g., parental monitoring) which influence video-gaming before bed time. Yet, videogame addiction is an important mechanism promoting this behavior.

*Pilot testing the FitBit Sleep Measurement*

The algorithm used to measure sleep duration is not released by Fitbit Inc. and the measurement cut-offs are unknown. Furthermore, it has not been frequently used in past research for measuring sleep. Therefore, a comparison between sleep duration measured by the Fitbit and sleep duration measured by the ActiSleep monitor (ActiGraph LLC, Pensacola, FL) was undertaken in five adults. Algorithms developed by Sadeh et al. [[2](#_ENREF_2)] were applied to score ActiSleep data. This algorithm has been previously validated against polysomnography with roughly 90% agreement and was tested in adults and adolescents. Sleep duration measured by the Fitbit highly correlated with sleep duration measured by ActiSleep using the Sadeh algorithm (Fitbit-Sadeh correlation of *r*=0.946, *p*<0.001). This has demonstrated the validity and reliability of Fitbit sleep measures.

S1 Text. Appendix C - Videogame Addiction Scale

While several studies suggest cutoffs for classifying people as addicts or not, the medical profession is still debating this issue [[3](#_ENREF_3)], and calls for further research before such determination can be made [[4](#_ENREF_4)]. Hence, consistent with this view and prior research in this realm [e.g., [5](#_ENREF_5),[6](#_ENREF_6)] we treated addiction as a continuous concept, ranging from low to high levels of addiction, rather than as a dichotomous concept. This prevents information loss due to no-yet substantiated dichotomization of addiction. The scale used is based on [[7](#_ENREF_7)] and is provided below:

In the last several months, how often….

- Do you find it difficult to stop using videogames when you are online or bored?
- Do you continue to use videogames despite your intention to stop?
- Do others (e.g., parents, siblings, friends) say you should use video games less?
- Do you prefer to use videogames instead of spending time with others (e.g., family, friends)?
- Are you exhausted because of videogames?
- Do you think about videogames, even when not online?
- Do you look forward to your next videogame session?
- Do you think you should use videogames less often?
- Have you unsuccessfully tried to spend less time on video games?
- Do you rush through your homework or chores in order to play videogames?
- Do you neglect your daily obligations (school, chores, family life) because you prefer to play videogames?
- Do you play video games when you feel down?
- Do you play videogames to escape from your sorrows, or get relief from negative feelings?
- Do you feel restless, frustrated, or irritated when you cannot play video games?

**References**

1. van den Eijnden RJJM, Spijkerman R, Vermulst AA, van Rooij TJ, Engels RCME (2010) Compulsive Internet Use Among Adolescents: Bidirectional Parent–Child Relationships. Journal of Abnormal Child Psychology 38: 77-89.

2. Sadeh A, Sharkey KM, Carskadon MA (1994) Activity-based sleep-wake identification: An empirical test and methodological issues. Sleep 17: 201-207.

3. Turel O, He Q, Xue G, Xiao L, Bechara A (2014) Examination of neural systems sub-serving Facebook "addiction". Psychological Reports 115: 675-695.

4. American Psychiatric Association (2013) Internet Gaming Disorder. Diagnostic and statistical manual of mental disorders (5th ed). 5th ed. Arlington, VA: American Psychiatric Publishing. pp. 795-798.

5. Byun S, Ruffini C, Mills JE, Douglas AC, Niang M, et al. (2009) Internet addiction: Metasynthesis of 1996-2006 quantitative research. Cyberpsychology & Behavior 12: 203-207.

6. Xu ZC, Turel O, Yuan YF (2012) Online game addiction among adolescents: motivation and prevention factors. European Journal of Information Systems 21: 321-340.

7. van Rooij AJ, Schoenmakers TM, Vermulst AA, van den Eijnden R, van de Mheen D (2011) Online video game addiction: identification of addicted adolescent gamers. Addiction 106: 205-212.
